# Primary versus Salvage Liver Transplantation after Curative-Intent Resection or Radiofrequency Ablation for Hepatocellular Carcinoma: Long-Term Oncological Outcomes

**DOI:** 10.3390/cancers15205030

**Published:** 2023-10-18

**Authors:** Alessandro Anselmo, Leandro Siragusa, Paolo Brigato, Camilla Riccetti, Andrea Collini, Bruno Sensi, Giuseppe Tisone

**Affiliations:** 1 Department of Surgical Sciences, Hepatobiliary and Transplant Unit, Policlinico Tor Vergata, 00133 Rome, Italy; 2 Department of Surgical Science, University of Rome “Tor Vergata”, 00133 Rome, Italypaolo.brigato@outlook.it (P.B.);; 3 Renal Transplant Center, Siena University Hospital, 53100 Siena, Italy

**Keywords:** liver surgery, hepatocarcinoma, downstaging, RFA, salvage transplant, transplant oncology

## Abstract

**Simple Summary:**

This research aimed to compare the outcomes of primary liver transplantation (PLT) and salvage liver transplantation (SLT) for hepatocellular carcinoma (HCC). The aim was to determine which approach offers better oncological outcomes and whether SLT after previous curative treatments such as liver resection (LR) or radiofrequency ablation (RFA) is as effective as PLT. Data analyzed from 141 patients who underwent liver transplantation for HCC found that PLT resulted in significantly longer disease-free survival, overall survival, and cancer-specific survival (CSS) compared to SLT. However, within the SLT group, there was no significant difference in DFS between SLT-LR and SLT-RFA. These findings suggest that PLT may provide superior long-term oncological outcomes, and a shift towards PLT rather than SLT may be worth considering. Further research is needed to confirm these results, but this study provides valuable insights for the research community regarding optimal transplantation strategies for HCC patients.

**Abstract:**

Liver transplantation for hepatocellular carcinoma (HCC) may be performed ab initio, primary liver transplantation (PLT), or for HCC recurrence after previous treatments such as liver resection (LR) or radiofrequency ablation (RFA), salvage liver transplantation (SLT). The aim of this study was to evaluate the oncological outcomes of SLT vs. PLT. For this, a retrospective study was carried out on patients undergoing liver transplantation for HCC. The outcomes of PLT were compared with those of SLT. The primary outcome was disease-free survival (DFS). The secondary outcomes included overall survival (OS), cancer-specific survival (CSS), and major postoperative complications. A sub-analysis of SLT-LR and SLT-RFA was also performed. In total, 141 patients were included: 96 underwent PLT and 45 SLT. Among the SLT group, 25 patients had undergone previous LR while 20 had had RFA. There were no differences in the major postoperative complications. Unadjusted DFS was significantly longer in the PLT group (*p* = 0.02), as were OS (*p* = 0.025) and CSS (*p* = 0.001). There was no difference in DFS between PLT and SLT-LR groups, while a significant difference was found between the PLT and SLT-RFA groups (*p* = 0.035). Nonetheless, DFS was no different between the SLT-LR and SLT-RFA groups. PLT appears to offer superior long-term oncological outcomes to SLT. Both SLT-LR and SLT-RFA offer acceptable OS and CSS. Further prospective studies are needed to confirm these results, but the re-direction of grafts and transplant philosophy towards PLT rather than SLT may need to be considered.

## 1. Introduction

Hepatocellular carcinoma (HCC) stands as a significant contributor to the global cancer burden, ranking as the third leading cause of cancer-related mortality, and is responsible for 830,200 deaths every year [1].

Treatments for HCC have evolved over time, and two potentially curative options include liver resection (LR) and radiofrequency ablation (RFA). While with small tumors RFA effectively represents a valid alternative to surgical treatment, for larger lesions, surgery remains the only treatment that can offer chances of cure [2].

Consequently, primary LR has emerged as the preferred treatment for early HCC in individuals with preserved liver function and mild portal hypertension [3]. The main surgical options in this context are LR and liver transplantation (LT). Nevertheless, despite its efficacy, the 5-year overall survival (OS) and 5-year disease-free survival (DFS) following LR for HCC reveal the challenges posed by cancer relapses, standing at up to 60% and 30%, respectively [4,5]. The oncological advantages of LT for HCC are well-documented, showcasing notable 5-year OS and 5-year DFS rates of up to 75% and 90%, respectively [6,7]. However, even among patients meeting the LT criteria, primary liver transplantation (PLT) is not invariably the preferred course of action. The shortage of available organs and the persistent risk of drop-out from the waiting list due to tumor progression and deteriorating liver function pose significant limitations to LT as a widespread solution [8].

Addressing the recurrence of HCC or the deterioration of liver function post primary liver resection, salvage liver transplantation (SLT) has emerged as an alternative and promising curative strategy. Originally proposed by Majno et al. in 2000, SLT is intended to be similar to LT following the recurrence or deterioration of the hepatic function after prior intent-to-cure treatment, such as radiofrequency ablation [9].

However, the oncological results of SLT are currently controversial, especially when compared to those of PLT [10,11,12]. Therefore, the aim of this study was to investigate the long-term oncological results of PLT compared to SLT.

## 2. Materials and Methods

This study included patients between 18 and 70 years of age who underwent LT for HCC at Policlinico Tor Vergata. Patients were identified retrospectively from a prospectively maintained database. This study was conducted according to the international ethical recommendations on clinical research established by the Helsinki and Istanbul Declarations. According to local institutional review board, ethical approval for retrospective studies is not required.

### 2.1. Patients

Patients with a diagnosis of HCC (according to American Association for the Study of Liver Diseases) undergoing LT were eligible for inclusion in this study [13]. Patients with any treatment or down-staging procedure were included. Patients beyond Milan or UpTo7 criteria were also included, either after appropriate down-staging or very individualized multidisciplinary indication for LT. Patients with recurrence after LR or RFA, non-susceptible to further tratments in absence of extra-hepatic disease, were considered for SLT. Patients without pathologically confirmed HCC diagnosis were excluded along with those who featured macroscopic vascular invasion, extrahepatic metastases, or concurrent malignancies. Re-transplantation patients were also excluded, as were patients without evidence of HCC recurrence in the interval between LR/RFA and LT.

All consecutive patients with available follow up operated on between 1 January 2011 and 31 December 2021 were evaluated for inclusion.

### 2.2. Study Design

Retrospective single-centre study comparing the short- and long-term outcomes of PLT and SLT.This study adhered to the Strengthening the Reporting of Observational Studies in Epidemiology (STROBE) Statement [14]. 

Patients who underwent PLT were compared with those who underwent SLT for HCC recurrence after LR or curative intent RFA. PLT was defined as LT in a patient with HCC and no prior curative-intent treatment. SLT was defined as LT in a patient with recurrent HCC after prior curative intent LR or RFA. Patients who underwent unique “bridging” therapies before LT, such as TACE or non-curative intent RFA (e.g., for lesions > 3 cm), were considered to have underwent PLT. Sub-analysis of outcomes of SLT after LR (SLR-LR) and after RFA (SLT-RFA) groups was also conducted.

HCC diagnosis was histologically confirmed on the surgical specimen. Indications for PLT, LR, or RFA and SLT were given at a focused multidisciplinary team (MDT) meeting including transplant surgeon, hepatologist, oncologist, radiologist, interventional radiologist, and pathologist. Surgery was performed following universally accepted transplantation and oncological principles at a specialized tertiary referral hepatobiliary unit. LR included both wedge and anatomical resections and choice was left to the operating surgeon. For LT, only cadaveric grafts were used. 

Immunosuppression (IS) was managed by the transplant team, with “standard starting therapy” being prolonged-release tacrolimus 4 mg once daily and everolimus 0.50 mg twice daily. Other used regimens included calcineurin-inhibitor (CI) sparing regimens with Basiliximab induction for patients with deterioration of renal function. Modifications of IS were delivered depending on individual IS through levels; usually 3–5 ng/mL for Everolimus and 5–9 ng/mL for Tacrolimus. Graft function was monitored weekly initially and then at gradually enlarging intervals, reaching every six months by the third year. IS minimization strategies were used liberally, usually favouring everolimus monotherapy for its anti-neoplastic effects.

Oncologic therapy was chosen by MDT following latest practice guidelines in an effort to provide best possible therapy for each patient. Oncologic follow up included physical examination, alpha-feto protein (AFP), CA 19–9, and thoraco–abdominal–pelvic CT every 6–12 months for the first 5 years.

### 2.3. Outcome Measures

Primary outcome of this study is DFS, defined as the length of time elapsed between date of LT and disease recurrence or death, whichever occurred first. Disease recurrence was defined as the appearance of new hepatic or extra-hepatic lesions or even elevated AFP levels alone.

Secondary outcomes were: OS, cancer-specific survival (CSS), and major postoperative complications. OS was defined as the length of time elapsed between date of LT and death from any cause. CSS was defined as the length of time elapsed between date of LT and death attributable to cancer. Major postoperative complications were defined as category ≥ IIIB according to Clavien–Dindo classification [15].

### 2.4. Study Variables

Pre-LT data included age, gender, American Society of anaesthesiologists (ASA) score, tumor characteristics (size, number, and location of lesions), MELD, MELD-Na, and Child–Pugh score. For the SLT group, primary procedure details were also recorded.

LT details included operative time, intraoperative complications, and intraoperative transfusions.

Postoperative data included postoperative complications, length of stay (LOS), histopathological report, and initial IS therapy. Follow up included HCC recurrence and mortality.

### 2.5. Statistical Analysis

Patients’ characteristics were summarized using cross-tabulations for categorical variables or using quantiles for continuous variables. In univariate analysis, non-parametric tests were performed for comparisons between groups (Chi Squared and Fisher Exact test in case of categorical variables or response rate, Mann–Whitney and Kruskal–Wallis test in case of continuous variables). Survival distributions have been estimated using the Kaplan–Meier Product Limit estimator. Cox regression models were used in univariate and multivariate analyses to assess the effect of the factors on survival outcomes (OS and DFS). Hazard Ratios (HR) and 95% Confidence Interval were reported as parameter results of the Cox regression models. Cancer specific mortality was analyzed in the framework of competing risk analysis. Cumulative incidence curves were reported for both cancer-specific and non-cancer-specific events. Groups were compared using Gray’s test. All tests were 2-sided, accepting *p* < 0.05 as indicating a statistically significant difference. All analyses were performed using R software (R Core Team (2021) R Foundation for Statistical Computing, Vienna, Austria).

## 3. Results

**Figure 1:**
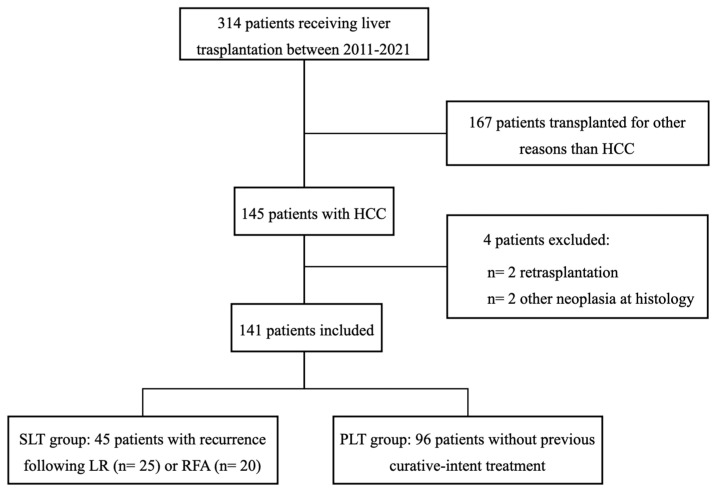
Patients selection.

A total of 314 patients underwent LT at “Policlinico Tor Vergata” between 2011 and 2021, 145 of which had a pre-operative HCC diagnosis. Four patients met the exclusion criteria, while 141 were included in the analysis (Figure 1). Ninety-six patients underwent PLT and 45 underwent SLT.

### 3.1. Patients’ Demographics

Patients in the PLT group had a significantly higher Child–Pugh (*p* = 0.0104) and MELD (*p* = 0.0206) score and were less likely to have undergone previous neo-adjuvant sorafenib therapy (5.2% vs. 17.7%; *p* = 0.0161) or abdominal surgery (53.1% vs. 77.7%; *p* = 0.0104).

Other characteristics were comparable, with no significant differences between groups.

The most prevalent underlying liver disease was HCV in both groups (44.8 vs. 60% in PLT and SLT respectively; *p* = 0.092). HCC lesions had similar characteristics, with 15.62% of PLT patients being out of Milan (vs. 13.3% of SLT patients; *p*= 0.921) and 4.0% out of the UpToSeven criteria (vs. 6.7% of SLT patients; *p*= 0.830), respectively. Bridging strategies were used in 70% and 68.9% of PLT vs. SLT patients, respectively (*p =* 0.913).

Among the SLT patients, 56% (*n =* 25) underwent LR and 44% *(n =* 20) underwent RFA. The first surgery characteristics are summarized in Table S1. A surgical approach was most often open (76%) and resection was anatomical (60%). R0 resection was obtained in 84% of patients. Eight percent (*n =* 2) had a second (R0) LR before LT. The mean interval between the first curative-intent treatment and recurrence was 19 ± 16.5 among the SLT group. The mean interval between the first curative-intent treatment and LT was 22.8 ± 18.5 among the SLT group.

The demographic and oncological characteristics of the two groups are summarized in Table 1 and Table 2, respectively.

### 3.2. Perioperative Outcomes

Intraoperative complications occurred in 7.2% vs. 8.9% in PLT vs. SLT, respectively (*p* = 0.741). No difference was detected in the 30-days’ major complications (27.10% after PLT and 28.9% after SLT; *p*= 0.8419). PLT patients required more postoperative transfusions than SLT patients (52.1% vs. 33.30%; *p*= 0.0373). LOS was also similar with a median of 9 (7–13) days in PLT and 8 (7–12) days in SLT (*p*= 0.4080). Perioperative outcomes are summarized in Table 3.

### 3.3. Survival

**Figure 2:**
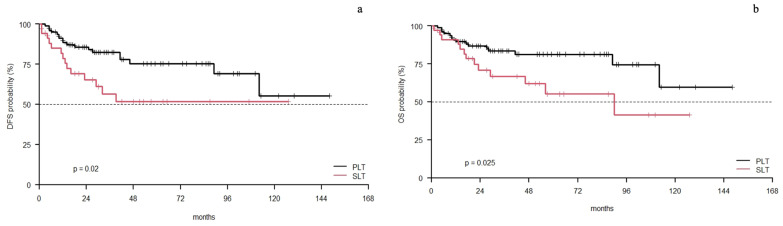
PLT vs. SLT Kaplan–Meier curves: (**a**) disease-free survival; (**b**) overall survival.

At a median follow up of 48 (1–148) months, 5.2% of patients (5/96) had HCC recurrence in the PLT group and 17.7% in the SLT group (8/45) (*p*= 0.02). The unadjusted DFS was significantly in favor of the PLT group (*p*= 0.02) (Figure 2a). The estimated DFS at the 1- and 3-year follow up was 88% (C.I. 82–96%) and 82% (C.I. 74–92%) in the PLT group, and 79% (C.I. 66–94%) and 56% (C.I. 41–78%) in the SLT group. In the SLT group, 4.4% received liver graft resection after LT, achieving R0 in all cases.

During the follow up, 32.3% of patients died after PLT and 51.1% after SLT. A significant difference was also noted in the unadjusted overall survival in the PLT and SLT groups (*p*= 0.025) (Figure 2b). The estimated overall survival at 1 and 3 years was 90% (C.I. 83–97%) and 84% (C.I. 76–93%) in the PLT group, and 91% (C.I. 82–100%) and 67% (C.I. 52–86%) in the SLT group.

**Figure 3:**
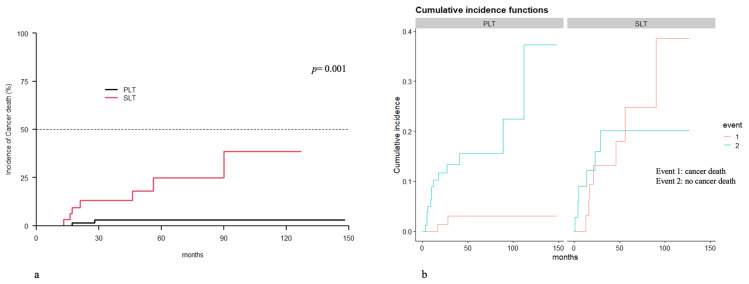
PLT vs. SLT cancer-specific survival: (**a**) incidence curve; (**b**) cumulative incidence curve.

Cancer-specific death occurred in 2.08% vs. 15.56% of PLT and SLT patients, respectively. The competitive risk analysis found cancer-specific survival to be significantly longer in the PLT group (*p* = 0.001) (Figure 3).

The univariate analysis found the transplantation setting (PLT vs. SLT) and AFP to be the only significant factors affecting OS, while DFS was affected by the same two factors and additionally by the MELD score. During the multivariate analysis, Cox models confirmed significant associations between the transplantation setting and AFP with both OS and DFS, and between the MELD score and DFS (Table 4 and Table 5).

### 3.4. SLT-LR vs. SLT-RFA: Patients’ Demographics

SLT-LR patients had a significantly lower MELD score (*p*= 0.019), a higher INR (*p* = 0.001), and were more likely to be Child–Pugh A (*p*= 0.011) and ex-alcoholics (*p*= 0.031). The median time to recurrence was no different amongst the subgroups.

Demographics of SLT-LR and SLT-RFA patients are summarized in Table S2.

### 3.5. SLT-LR vs. SLT-RFA: Perioperative Outcomes

There were no differences in the perioperative outcomes except for postoperative blood transfusions, which were less required in SLT-LR patients (*p* = 0.034) (Table S3).

### 3.6. SLT-LR vs. SLT-RFA vs. PLT: Survival

**Figure 4:**
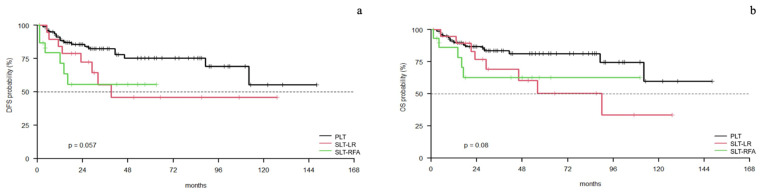
PLT vs. R vs. RFA Kaplan–Meier curves: (**a**) disease-free survival; (**b**) overall survival.

Unadjusted DFS was significantly improved in the PLT group compared to the SLT-RFA group (*p*= 0.035), while there was no difference between the PLT and SLT-LR (*p* = 0.0720) or LR and RFA groups (*p*= 0.7740) (Figure 4a). The estimated DFS at the 1- and 3-year follow up was 88% (C.I. 82–96%) and 82% (C.I. 74–92%) in the PLT group, 84% (C.I. 69–100%) and 55% (C.I. 34–89%.) in the SLT-LR group, and 72% (C.I. 51–100%) and 56% (C.I. 34–90%) in the SLT-RFA group. During the follow up, 32.3% of patients died after PLT, 50.0% after SLT-LR, and 52% after SLT-RFA. There was no difference in the unadjusted OS between PLT and SLT-RFA (*p*= 0.0830), PLT and SLT-LR (*p*= 0.0600), nor SLT-LR and SLT-RFA (*p*= 0.907) (Figure 4b). The estimated overall survival at 1 and 3 years was 90% (C.I. 83–97%) and 84% (C.I. 76–93%) in the PLT group, 95% (C.I. 85–100%) and 6% (C.I. 49–86%) in the SLT-LR group, and 86% (C.I. 70–100%) and 63% (C.I. 41–95%) in the SLT-RFA group.

**Figure 5:**
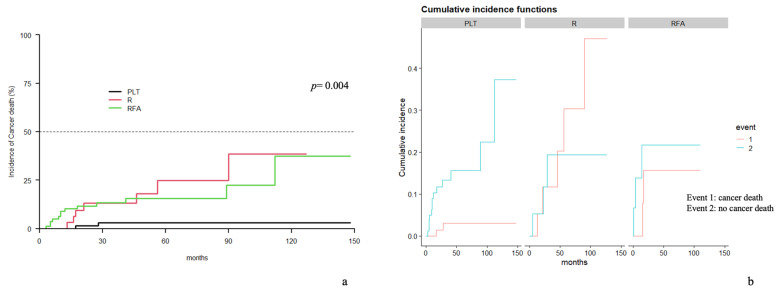
PLT vs. R vs. RFA cancer-specific survival: (**a**) incidence curve; (**b**) cumulative incidence curve.

Competitive risk analysis found cancer-specific survival to be significantly longer in the PLT group (*p* = 0.004). (Figure 5). In the univariate analysis, SLT-RFA seemed to be correlated with DFS while SLT-LR was not. However, both were associated with a shorter DFS in the multivariate analysis (Table 3).

## 4. Discussion

The current study compared the short- and long-term outcomes of patients undergoing PLT with those undergoing SLT for HCC. Disease-free survival was significantly longer in the PLT group compared to the SLT group, after a median of a 48-month follow up. Other main oncologic outcomes, including OS and CSS survival were also significantly in favor of the PLT group. The LT setting (primary vs. salvage) was confirmed in the multivariate analysis to be an independent predictor of disease recurrence and death.

LT represents the most effective treatment for HCC, although it is associated with having a high perioperative risk and is generally life-long IS [16,17]. Ideally, PLT would probably be the best option for most patients. SLT is an oncological strategy for the management of HCC that contemplates the use of LT only after the failure (HCC recurrence) of a different, primary, curative-intent procedure. This option was developed in an effort to minimize the use of grafts for this indication, which generally carries a poorer prognosis compared to most benign indications [18].

The literature on SLT is abundant, but no study to date carries a high level of evidence (i.e., there are no randomized studies) and many refer to patients treated back in the beginning of the century. In general, conclusions have been conflicting with studies supporting a survival advantage for both operations or no differences at all. Two large meta-analyses support a survival advantage for SLT in both OS and DFS, especially for the recipients of cadaveric grafts [19,20]. However, both studies are highly biased by the fact that they have included in the SLT group patients that underwent LT not for HCC recurrence, but for eventual end-stage liver failure [19,20]. These patients are no help in recognizing the oncological impact of LT timing. While results of SLT remain controversial [11,21,22], many other studies on the matter have reported a superior survival with PLT, in line with this present study [23,24,25,26]. The largest study, by Hu et al., on 6975 patients found PLT to lead to superior DFS [23].

Of note, many studies, rather than comparing PLT with SLT, compare PLT with an organ-sparing strategy that makes use of SLT [9,27]. In these studies, survival was calculated from the first curative-intent procedure, with some HCC patients that were not transplanted due to the achievement of a cure with the primary procedure, while others also were never transplanted due to the recurrence of falling out of the transplantation criteria. For the former patients, PLT might have represented an over-treatment, while for the latter, its withholding signified condemnation. In fact, the transplantability rate for recurrence is generally low, varying from 25% to 52% [24,27,28]. These studies compare the overall management strategy, but do not provide meaningful information on the efficacy of the SLT procedure, per se.

In this present study, the overall strategy has been ignored and survival was calculated from the moment of SLT. This permitted the direct comparison of the results of the LT procedure itself and the conclusion seems clear: grafts are better spent on PLT than SLT. In this perspective, SLT may be a sub-optimal solution even from the organ-shortage perspective, as liver grafts are being destined (and “wasted”?) to patients who are much less likely to benefit from them.

Of course, the choice of this particular timeline may also be regarded as a limitation of this study, which in fact provides important, novel data on the SLT procedure, but less so from a broader, SLT strategy point of view.

A main bias of all SLT studies is that patients who have been assigned to the PLT group were less fit and could not have undergone the SLT strategy anyway, as their liver function would not permit any other procedure without taking very high risks. In fact, even in this present study, SLT patients had better liver function, and this difference would have been even more radical when considering these patients’ status at their first curative intent procedure time-point. Therefore, this difference in the general status and liver function especially represents an even larger bias in studies considering the overall SLT strategy.

Multivariate analysis outlined other important findings. One factor independently associated with DFS and OS was AFP, reflecting the importance of HCC biology on the final outlook. In fact, recent studies have suggested the importance of incorporating AFP levels into LT listing criteria [29]. The MELD score was only associated to OS and not to DFS, indicating how liver function is indeed fundamental in-patient survival, but does not influence the occurrence of recurrence. The Milan and up-to-7 criteria were not associated with OS and DFS, probably due to an accurate selection of patients and a limited number of patients outside these criteria.

Major complications were similar among groups. This may appear surprising as SLT is performed after a previous invasive liver procedure that is expected to produce adhesions and render surgery more difficult. In fact, early in the SLT experience, some authors have indeed found an increase in postoperative complications for SLT [24]. Lately, the use of laparoscopic resection may have facilitated SLT [30]. Overall, the present results, along with other reports, testify to the (relative) safety of SLT. In fact, the only postoperative difference among groups was a higher use of transfusion in the PLT group, probably ascribable to the initial higher MELD score.

Of note, this study is one of the first studies to consider SLT after any curative-intent procedure including, therefore, both surgery and RFA. This is justified in that RFA under certain circumstances (taken into consideration in the inclusion criteria) can be considered a curative treatment with oncological results similar to those of surgery [2]. In fact, the SLT-RFA strategy in HCC patients within the Milan criteria and with Child–Pugh A cirrhosis has been reported to lead to an outstanding 69% 5-years DFS rate, which can be considered at least comparable to the DFS rates reported for SLR-LR [31].

Nonetheless, a sub-analysis of SLT-LR vs. SLT-RFA was performed both to compare the results of SLT after different curative treatments and to identify potential bias. The results of this analysis are interesting, highlighting an improved DFS for PLT compared to SLT-RFA only, but no difference between SLT-RFA and SLT-LR. OS was similar amongst groups, while CSS was longer in the PLT group compared to both SLT-RFA and SLT-LR. These results suggest a possible survival advantage with the surgical option rather than the radiological one, yet this must be balanced against a better base-line liver function and increased neoadjuvant sorafenib use in the SLT-LR group. In fact, the CSS was not different between the SLT strategies. Muaddi et al. have recently reported on their experience with SLT after both RFA and LT, similarly to this present study, and their findings were essentially in-line with those from this present study in as much that SLT-RFA and SLT-LR had similar OS results [11]. Differently, in their study, SLT was comparable to PLT in terms of recurrence when case-matched analysis was undertaken [11].

The current study carries some limitations. The main bias of this study is that patients in the SLT group are, by definition, a high-risk population, as they have all experienced disease recurrence. It is possible that HCC in the SLT group had an intrinsically more aggressive biological behaviour, therefore skewing the results toward a shorter survival. Nonetheless, AFP levels, which for the moment are considered one of the most important parameters of biological behaviour, were similar between the groups. The length of the follow up (median of 48 months) may be regarded as a weakness of the analysis given that the “standard” 5 years were not reached by many patients, yet it was sufficiently long for important differences to emerge, and it is likely that a longer follow up would have strengthened these differences. Therefore, a follow up could still be considered adequate. Other limitations include a relatively small sample size, the retrospective design, and the monocentric nature of this study, limiting the generalizability of results. Another limitation may be related to the use of extended donor criteria organs that could bias perioperative results but should not have an influence on DFS and OS. The single-center design may be regarded also as a strength in that uniformity in management, including pre-, intra-, and post-LT care, was guaranteed.

The number of patients in the PLT group was more than twice as many as those in the SLT group, potentially representing a source of bias. On the other hand, this is also indicative of the choices made by the multidisciplinary transplant team. It is clear that in the new scenario, which emerged in the past decade, where HCV has become a highly curable disease, more and more grafts will be dedicated to transplant oncology. In fact, HCV was among the most prevalent indications for LT, representing 12% of them in 1996–2006, while with the advent of Direct Acting Antivirals (DAAs), eradication is now possible for 69–91% of cases and LTs for HCV have been drastically decreasing (halved to 6% of LTs) [32,33,34]. At the same time DAAs do not appear to have diminished HCV-related HCC development and LTs performed for HCC have doubled from 12–24% lately [32,34]. An organ shortage seems to be a less impacting problem nowadays, giving transplant teams the liberty to re-direct many more livers to oncologic patients. Additionally, Italy’s current blend facilitates easier access and shorter waiting times for HCC patients on the transplant list [35]. Another significant contributor to an expanded organ pool is the growing adoption of systematic machine perfusion. This enhances the availability of extended donor criteria organs for transplantation, which are often the most used in the case of HCC patients. Other factors augmenting the organ pool is now the more systematic use of machine perfusion. Some groups have gone so far in the organ sparing philosophy as to propose a second resection after HCC recurrence. This study has not considered this approach, yet results for this strategy have been shown to be inferior to SLT [12,36,37,38]. Rather than organ-sparing, it is probable that the time has come to shift our efforts to delivering the most effective oncological treatment upfront in an effort to maximize survival outcomes. This direction appears to be the best solution, indeed, opening for the better employment of liver grafts given the superior results of PLT compared to SLT. New tools for an accurate prediction of the outcome after PLT versus primary LR or RFA are needed to better select the optimal candidates for the PLT strategy [39]. At the same time, patients who undergo LR and are found to be at high risk for recurrence may undergo a “sequential” LT strategy, including pre-emptive LT before HCC recurrence. This strategy still needs to be investigated further as these “high-risk” patients are not well delineated yet [40,41]. On this line, living donor SLT is an approach that may further expand the liver graft pool and further push the PLT strategy. Many studies performed in the east have demonstrated the feasibility and similarity of results to deceased donor SLT [20,22,42,43]. Barriers to worldwide implementation are, for the time being, limiting the impact of this approach.

Another limitation of this study is that most LTs in this series were performed with the piggy-back technique, while the “traditional” LT technique represents a “no-touch” technique, theoretically affording larger oncological guarantees. Nonetheless living donor studies (where piggy-back is the only option) have suggested the oncological equivalence of the two surgical techniques [22,42,43,44].

The two groups had very similar pre-operative characteristics, most notably in the HCC burden, represented by the Milan and Up-to-seven criteria, emphasizing the reliability of the oncological outcomes. There were few significant differences. These differences included a decreased prevalence of previous abdominal surgery and neoadjuvant sorafenib therapy in the PLT group, along with a higher Child–Pugh and MELD score. While the former is an intuitive result of the two management strategies, the latter probably represent conscious patient selections, with the fittest patients undergoing an SLT strategy while patients unfit for surgery or even RFA received PLT. Obviously, this represents a meaningful bias: an inferior liver function and the overall general conditions in the PLT group may have increased the mortality in this group. This bias should have conferred a survival advantage to the SLT group, which resulted in having a shorter DFS, OS, and CSS anyway; therefore, from a different perspective, this may possibly increase or, at least, confirm the robustness of the results obtained. This also seems to suggest that selecting fitter patients for PLT rather than SLT could realistically further enhance outcome progress.

Another strength of our study was the investigation of cancer-specific survival in the framework of competing risk analysis, given that, as said, PLT patients are generally more organ-compromised and thus more likely to suffer death from other causes. This analysis has not been performed, to the best of our knowledge, in other similar studies and helps to shed light on SLT oncologic results, eliminating substantial bias.

## 5. Conclusions

PLT appears to carry superior DFS, OS, and CSS compared to SLT despite being offered to patients with more profoundly compromised organs. SLT-RFA and SLT-LR may not differ in their oncological outlook. A prospective and randomized study is needed to confirm the findings of this present study. Meanwhile, the re-direction of grafts and transplant philosophy towards PLT rather than SLT may need to be considered.

## Figures and Tables

**Table 1:** PLT vs. SLT demographics characteristics. Bolds means statistical significancy.

Parameters	SLT (n = 45)		PLT (n = 96)		*p*
Age (mean, SD)	57.9 ± 7.47		58.3. ± 6.5		0.719
Sex (n, %)					0.628
Male	39	86.6%	79	82.3%	
Female	6	13.4%	17	17.3%	
Preoperative BMI (kg/m^2^) (mean, SD)	26.2 ± 4.7		26.9 ± 4.2		0.390
ASA score (n, %)					0.749
1	1	2.2%	0	0%	
2	3	6.6%	11	11.4%	
3	33	73.3%	71	73.9%	
4	8	17.7%	14	14.6%	
Smoker (n, %)	16	35.6%	30	31.25%	0.700
Ex-Alcoholic (n, %)	26	57.7%	40	41.6%	0.102
Previous abdominal surgeries (n, %)	35	77.7%	51	53.1%	**0.005**
	10	22.3%	45	46.9%	
Comordities. (n, %)					
Diabetes	16	35.5%	40	41.6%	0.489
Hypertension	22	48.9%	47	48.9%	0.993
Cardiovascular disease	0	0%	7	7.3%	0.437
Respiratory disease	1	2.2%	4	4.16%	0.560
Previous neoplasm	2	4.4%	2	2.1%	0.431
Underlying liver disease (n, %)					
HBV	6	13.3%	10	10.4%	0.610
HBV + HDV	2	4.4%	4	4.1%	0.939
HCV	27	60%	43	44.8%	0.092
Alcohol	7	15.5%	19	19.8%	0.545
NASH	3	6.6%	17	17.7%	0.079
Other	0	0%	3	3.1%	0.748
Child–Pugh Score (n, %)					**0.01**
A	35	77.7%	49	51%	
B	8	17.7%	36	37.5%	
C	2	4.4%	11	11.46%	
MELD Score (mean, DS)	10.4 ± 4.		12.3 ± 4.7		**0.02**
Albumin (gr/dL) (mean, SD)	3.5 ± 0.6		3.2 ± 0.5		0.620
Hemoglobin (gr/dL) (mean, SD)	12.3 ± 2.1		11.6 ± 2.1		0.425
Bilirubin (mg/dL) (mean, SD)	1.3 ± 1.5		2.4 ± 4.1		0.570
PT-INR (mean, SD)	1.3 ± 0.2		1.4 ± 0.3		0.133
Creatinine (mg/dL) (mean, SD)	0.9 ± 0.2		1 ± 0.5		0.248

**Table 2:** PLT vs. SLT oncological characteristics. Bolds means statistical significancy.

Parameters	SLT * (n = 45)		PLT (n = 96)		*p*
Alpha-fetoprotein (ng/mL) (mean, SD)	39.2 ± 93		47.2 ± 217.2		0.684
Milan criteria (n, %)					0.921
In	39	86.7%	81	84.4%	
Out	6	13.3%	15	15.6%	
Up to 7 (n, %)					0.830
In	42	93.3%	92	95.8%	
Out	3	6.7%	4	4.1%	
Number of nodules (mean, SD)	1.9 ± 0.9		1.9 ± 1		0.791
Size of largest nodule (mm) (mean, SD)	23.1 ± 10.2		26 ± 12.6		0.174
Bridging (TACE) (n, %)	31	68.9%	67	70%	0.913
Neo-adiuvant therapy (sorafenib) (n, %)	8	17.7%	5	5.2%	**0.016**
Interval between last radiologic treatment and LT (months) (mean, SD)	4.3 ± 4.1		4.5 ± 3.8		0.791

* SLT oncological characteristics refers to recurrence.

**Table 3:** PLT vs. SLT perioperative outcomes. Bolds means statistical significancy.

Parameters	SLT (n = 45)		PLT (n = 96)		*p*
Time awaited in list (days) mean, SD (median)	94.4 ± 104.2 (51)		103.7 ± 102.2 (84)		0.618
Associated surgeries (n, %)	2	4.4%	5	5.2%	0.845
Operative time (minutes) (mean, SD) (median)	477 ± 86 (470)		474.2 ± 89.9 (460)		0.860
Intraoperative complications (n, %)	4	8.9%	7	7.2%	0.741
Cardiac arrest	2	4.4%	2	2%	0.431
Bleeding	1	2.2%	2	2%	0.957
Absence of arterial pulse	2	2.2%	3	3.1%	0.692
Intraoperative transfusion (n, %)	34	75.5%	75	78.1%	0.734
Postoperative complications (n, %)	24	53.3%	54	56.2%	0.745
Anemia	13	28.9%	39	40%	0.178
Infection	11	24.4%	22	22.9%	0.841
Pleural effusion	2	4.4%	9	9.4%	0.308
Postoperative transfusion (n, %)	15	33.3%	50	52.1%	**0.037**
Postoperative complications (Clavien–Dindo)					0.913
None	0	0%	0	0%	
Minor (1-3a)	32	71.1%	70	72.9%	
Major (3b-5)	13	28.9%	26	27.1%	
Hospitalization, (days) (mean, SD) (median)	11.6 ± 15.4 (8)		13.9 ± 14.4 (9)		0.408
Tumor differentiation (G) (n, %)					0.066
1	3	6.6%	6	6.25%	
2	11	24.4%	30	31.25%	
3	22	48.9%	31	32.3%	
4	9	20%	13	13.5%	
Not available	0	0%	16	16.6%	
Microvascular invasion (n, %)	2	4.4%	6	6.2%	1.000

**Table 4:** Univariate and multivariate analysis of DFS. Bolds means statistical significancy.

Characteristic	Univariate			Multivariate		
	HR ^1^	95% CI ^2^	*p*-Value	HR ^1^	95% CI ^2^	*p*-Value
**Age**	1	1.00, 1.00	0.5			
**Sex**	0.8	0.37, 1.53	0.44			
**Group**						
** PLT**	—	—		—	—	
**SLT-LR**	2.1	0.89, 4.73	0.09	2.4	1.01, 5.85	**0.049**
**SLT-RFA**	2.5	1.00, 6.42	**0.05**	3	1.16, 7.80	**0.023**
**SLT**	2.2	1.11, 4.50	**0.024**	2.7	1.26, 5.56	**0.01**
**MELD score**	1	0.98, 1.11	0.15	1.1	1.01, 1.15	**0.017**
**Milano Criteria**	1	0.46, 2.13	0.98			
**Up-To-7**	3.1	0.43, 22.7	0.26			
**AFP**	1	1.02, 1.07	**<0.001**	1	1.01, 1.06	**0.002**
**Hb**	1	0.83, 1.09	0.5			
**Bilirubin**	1	0.93, 1.08	0.97			
**INR**	1.2	0.47, 2.88	0.75			
**Creatinine**	1	0.47, 2.01	0.95			
**Albumin**	0.6	0.36, 1.08	0.094			
**DM2**	1.1	0.58, 1.95	0.84			
**IA**	1.6	0.87, 2.81	0.14			
**Cardiovascolar**	0	0.00, Inf	>0.99			
**Respiratory**	1.7	0.41, 7.03	0.46			
**BMI (pre-tx)**	1	0.92, 1.06	0.79			
**ASA score**	1.1	0.64, 1.84	0.75			
**Previous abdominal surgery**	1.1	0.56, 2.04	0.84			

Abbreviations: ^1^ HR = Hazard Ratio, ^2^ CI = Confidence Interval.

**Table 5:** Univariate and multivariate analysis on OS. Bolds means statistical significancy.

Characteristic	Univariate			Multivariate		
	HR ^1^	95% CI ^2^	*p*-Value	HR ^1^	95% CI ^2^	*p*-Value
**Age**	1	1.00, 1.00	0.34			
**Sex**	0.71	0.34, 1.48	0.36			
**Group**						
**PLT**	—	—		—	—	
**SLT-LR**	2.23	0.94, 5.25	0.068	1.99	0.83, 4.75	0.12
**SLT-RFA**	2.41	0.87, 6.67	0.089	2.37	0.86, 6.55	0.1
**SLT**	2.29	1.09, 4.83	**0.029**	2.12	1.00, 4.49	**0.05**
**MELD score**	1.03	0.97, 1.10	0.35			
**Milano Criteria**	1.12	0.47, 2.67	0.8			
**Up-To-7**	0.0	0.00, Inf	>0.99			
**AFP**	1.04	1.01, 1.06	**0.003**	1.03	1.01, 1.06	**0.01**
**Hb**	0.96	0.83, 1.11	0.6			
**Bilirubin**	0.99	0.90, 1.10	0.89			
**INR**	0.94	0.34, 2.62	0.9			
**Creatinine**	0.97	0.44, 2.12	0.94			
**Albumin**	0.77	0.44, 1.33	0.35			
**DM2**	1.05	0.55, 2.01	0.87			
**IA**	1.69	0.90, 3.17	0.1			
**Cardiovascolar**	0	0.00, Inf	>0.99			
**Respiratory**	2.42	0.58, 10.1	0.23			
**BMI (pre-tx)**	0.98	0.91, 1.06	0.69			
**ASA score**	0.96	0.55, 1.69	0.89			
**Previous abdominal surgery**	1.18	0.59, 2.37	0.63			

Abbreviations: ^1^ HR = Hazard Ratio, ^2^ CI = Confidence Interval.

## Data Availability

Data underlying this article cannot be shared publicly due to the privacy of the individuals that participated in this study. The data may be shared on reasonable request to the corresponding author.

## Supplementary Materials

The following supporting information can be downloaded at: https://www.mdpi.com/article/10.3390/cancers15205030/s1, Table S1: Index liver surgery characteristics among R patients; Table S2: SLT-LR vs. SLT-RFA baseline characteristics; Table S3: SLT-LR vs. SLT-RFA perioperative outcomes.
