# Supplementary material for: Primary versus Salvage Liver Transplantation after Curative-Intent Resection or Radiofrequency Ablation for Hepatocellular Carcinoma: Long-Term Oncological Outcomes
Source: Cancers (Basel). 2023 Oct 18;15(20):5030. doi: 10.3390/cancers15205030 (PMC10605470; doi:10.3390/cancers15205030)
Supplement: Supplementary file 1 [file cancers-15-05030-s001.zip › cancers-2542977-supplementary.pdf]

Supplementary materials

Table S1: First liver surgery characteristics among R patients

Parameters	Group R (n=25)
Type of liver resection (n, %)	
Segmentectomy	13 52%
Bisegmentectomy	2 8%
Single wedge	7 28%
Double wedge	2 8%
Triple wedge	1 4%
Surgical approach (n, %)	
Laparoscopy	6 24%
Open	19 76%
Total operative time (minutes) (mean, SD)	192.2±38.8
Associated surgeries (n, %)	8 32%
Clavien-Dindo (n, %)	
1	21 84%
2	2 8%
3a	1 4%
3b	1 4%
Hospitalization (days)(mean, SD) (median)	7.5±3.4 (7)
Tumor differentiation (G) (n, %)	
1	3 12%
2	12 48%
3	7 28%
4	2 8%
Not detectable	1 4%

Table S2: Baseline characteristics: SLT-LR vs SLT-RFA

Parameters	SLT-LR (n=25)	SLT-RFA (n=20)	P
Age (mean, SD) (median)	57.7±8.2 (60)	58.2±6.6 (59,5)	0.826
Sex (n, %)			
Male	22 88%	17 85%	0.768
Female	3 12%	3 15%	
Preoperative BMI (kg/m^2) (mean, SD) (median)	26.2±4.2 (26)	26.2±5.3 (26)	1.000
ASA score (n, %)			
1	1 4%	0 0%	0.321
2	2 8%	1 5%	
3	20 80%	13 65%	
4	2 8%	6 30%	
Smoker (n, %)	10 40%	6 30%	0.486
Non smoker (n, %)	15 60%	14 70%	
Ex Alcoholic (n, %)	18 72%	8 40%	**0.031**
Non Alcoholic (n, %)	7 28%	12 60%	
Comordities. (n %)			
Diabetes	9 36%	7 35%	0.944
Hypertension	14 56%	8 40%	0.172
Cardiovascular disease	0 0%	0 0%	0.871
Respiratory disease	0 0%	1 5%	0.871
Previous neoplasia	2 8%	0 0%	0.688
Underlying liver disease (n, %)			
HBV	5 20%	1 5%	0.141
HBV + HDV	1 4%	1 5%	0.871
HCV	14 56%	13 65%	0.540
Alcohol	4 16%	3 15%	0.926
NASH	1 4%	2 10%	0.422
Other	0 0%	0 0%	0.871
Child-Pugh Score No (n, %)			
A	24 96%	11 55%	**0.011**
B	1 4%	7 35%	
C	0 0%	2 10%	
MELD Score (media, DS)	9.2±2.5	12±5	**0.019**
Alpha-fetoprotein (ng/ml) (mean, SD)	52.2±115.6	22±48	0.28

Albumin (gr/dl) (mean, SD)	3.7±0.6	3.4±0.6	0.102
Hemoglobin (gr/dl) (mean, SD)	12.8±1.9	11.6±2.2	0.056
Bilirubin (mg/dl) (mean, SD)	0.93±0.6	1.77±2	0.053
PT - INR (mean, SD)	1.16±0.12	1.37±0.28	**0.001**
Creatinine (mg/dl) (mean, SD)	0.9±0.2	0.84±0.2	0.322
Milan criteria (n, %)			
In	22 88%	17 85%	0.946
Out	3 12%	3 15%	
Up to 7 (n, %)			0.911
In	23 92%	19 95%	
Out	2 8%	1 5%	
Number of nodules (mean, SD)	2±0.9	1.8±0.9	0.462
Size of largest nodule (mm) (mean, SD)	22.1±10	24.3±10.7	0.481
Bridging (TACE) (n, (%)	18 72%	13 65%	0.614
Neo-adiuvant therapy (sorafenib) n, (%)	6 24%	2 10%	0.222
First recurrence's mean time (months) (mean, SD) (median)	19.3±20.5 (8)	10.9±11.4 (7)	0.108
Interval between curative intent treatment and LT (months) (mean, SD)	26.2±19.9	18.7±16.6	0,184
Interval between last radiologic treatment and LT (months) (mean, SD)	3.2±2.9	5.5±4.9	0.057

Table S3: Perioperative outcomes SLT-LR vs SLT-RFA

**Parameters**	**Group SLT-LR (n=25)**	**Group SLT-RFA (n=20)**	**P**
**Time await in list (days) (mean, SD) (median)**	83±85 (42)	109±125 (65)	0.412
**Associated surgeries (n, %)**	1 4%	1 5%	0.871
**Operative time (minutes) (mean, SD) (median)**	479.6±86.9 (480)	473.7±86.8 (465)	0.821
**Intraoperative complications (n, %)**	2 8%	2 10%	
**Cardiac arrest**	2 8%	0 0%	0.814
**Bleeding**	1 4%	0 0%	0.688
**Absence of arterial pulse**	0 0%	2 10%	0.871
			0.422
**Intraoperative transfusion (n, %)**	17 68%	17 85%	0.187
**Postoperative complications (n, %)**	12 48%	12 60%	0.422
**Anemia**	5 20%	8 40%	0.141
**Infection**	5 20%	6 30%	0.437
**Pleural effusion**	2 8%	0 0%	0.688
**Postoperative transfusion (n, %) (mean, SD)**	5 20%	10 50%	**0.034**
	1.2±3.6	3.1±5.6	0.175
**Postoperative complications (Clavien-Dindo)**			
**None**	0 0%	0 0%	0.796
**Minor (1-3a)**	19 76%	13 65%	
**Major (3b-5)**	6 24%	7 35%	
**Hospitalization, (days) (mean, SD) (median)**	7.3±4.3 (7)	15.3±21.7 (8.5)	0.07
